# The challenge of home and community older adult care in China: a survey of the capability of primary care physicians in providing geriatric healthcare services

**DOI:** 10.3389/fpubh.2024.1464718

**Published:** 2024-12-23

**Authors:** Xiufang Chen, Kun Xie, Yahui Li, Dan Hu, Yong Chen, Jiaying Chen

**Affiliations:** ^1^Kangda College of Nanjing Medical University, Lianyungang, China; ^2^Center for Health Policy Studies, Nanjing Medical University, Nanjing, China; ^3^Xuzhou Center for Disease Control and Prevention, Xuzhou, China; ^4^China Rural Health Association, Beijing, China

**Keywords:** home-based older adult care, PCPs, diagnostic capability, chronic illness, health management

## Abstract

**Introduction:**

In the context of ageing at home and in the community, healthcare services for older adult people in China are mainly provided by primary care physicians (PCPs). This study aimed to understand the level of geriatric healthcare service capability of PCPs in China and to give recommendations for improving care.

**Methods:**

We surveyed PCPs in the eastern, central, and western regions of China, using a combination of multi-stage stratified cluster sampling and typical sampling. We evaluated the capability of PCPs in geriatric healthcare services in terms of diagnosis and treatment of common diseases, health management of the older adult, and health management of patients with chronic diseases. We compared the capability of PCPs in different regions, in urban and rural areas, and with different characteristics.

**Results:**

We found that Chinese primary care doctors had a low level of education and tended to be older in age. We also found a lack of general practitioners in China. Among the 8,469 respondents, 6,497 (76.7%) could diagnose and treat common diseases; 7,755 (91.6%) were capable of managing the health of the older adult, and 7,787 (91.9%) could manage the health of patients with chronic diseases.

**Conclusion:**

Results indicated that China’s primary care physician workforce was of low quality. There were deficiencies in all competencies in geriatric healthcare services, and there were differences in capability between urban and rural areas. The findings suggest China’s primary healthcare services should be strengthened with targeted training and an emphasis should be placed on developing basic skills in geriatric healthcare services.

## 1 Introduction

In 2022, the global population of people aged 65 years old and above reached 970 million, accounting for about 10% of the world’s total population. The figure is expected to reach 1.6 billion by 2050, which will account for 16.5% of the total population (1). As the older adult population increases, the proportion of health issues in the overall population will be dominated by those diseases related to ageing, and the demand for geriatric services is expected to rise rapidly, as confirmed by studies around the world (2–4).

In response to the rising demand for healthcare services brought about by population ageing, the international community has actively advocated for and implemented a series of strategic planning and action measures. Healthy ageing and active ageing have become the focus of international actions to cope with this demographic change and its effects (5–7). In 2016, the Global Strategy and Plan of Action on Ageing and Health (2016–2020), adopted by the World Health Organization’s (WHO) 69th World Health Assembly, called on member states to achieve the development goal of healthy ageing by, among other things, promoting health systems responsive to the needs of the older population (8). In 2020, the WHO called on member states to provide health services that were responsive to the needs of the older population (9).

The issue of ageing and health has become a strategic focus for all countries, which have prioritized establishing and improving the geriatric health service system (10–12). In Japan, for example, in the context of ageing and a shortage of physicians, general practice has been identified as the 19th specialty in the field of clinical medicine in the new specialist physician system launched in 2018, and internists were encouraged to become general practitioners (GPs) (13). The United Kingdom has supplemented its GP workforce by recruiting foreign doctors and has established a long-term health service relationship with the older adult through family doctors to provide them with continuous medical services and health guidance (14, 15). In Australia, in the context of increased demand for healthcare services for the older adult, GPs have been trained for health monitoring skills and health data analysis. Most of the health problems of the older adult are chronic, requiring continuous and long-term care. Therefore, the primary services required are basic medical services and health management services (16, 17). PCPs are the main healthcare providers (18–21), and studies have concluded that they could undertake most healthcare services for the older adult population, such as diagnosis and treatment of common diseases, health management for the older adult and patients with chronic diseases (22–25). The PCP workforce is, therefore, actually crucial for the realization of the goals of healthy and active ageing.

By the end of 2022, 14.9% of China’s population was 65 years old or above, and China is expected to become a super-ageing society by 2030. In 2013, to address this issue, the Chinese government proposed the model of integrated eldercare services with healthcare. The aim was to integrate healthcare and life care services to meet the needs of older adult people for both health and daily life. The Healthy China 2030 strategy also requires integrating eldercare and healthcare services, strengthening health monitoring and comprehensive intervention for older adult people and chronically ill patients (26).

Based on traditional Chinese cultural customs and the desire of older adult people to choose their own way of ageing, the Chinese government established the “9,073” concept to guide development of the older adult care system, i.e., 90% of the older adult living at home, 7% cared by community facilities, and 3% living at nursing home. This means that about 97% of older adult people need daily life and healthcare services at grassroots level. In order to provide better services to those older adult people staying at home and community centers, the Chinese government has made relevant policies to ensure older adult care services provision, and many social organizations are providing daily life care services to those older adult people (27). However, healthcare needs are often quite high among the older adult, and should mainly rely on primary health providers. Previous studies have confirmed that the diagnosis and treatment of common diseases, health management of the older adult, and chronic disease management are the most important care to the older adult people (17–19). Therefore, it is necessary to enhance the health service capability of PCPs to provide professional health services to older adult people (28). As the cornerstone of China’s medical and health system, primary health organizations are responsible for providing primary healthcare to urban and rural residents. These organizations include community health service centers, community health service stations, township health centers, and village clinics.

China has historically prioritized hospitals and neglected the grassroots medical institutions in allocating health resources, resulting in a series of problems such as insufficient numbers, low quality, and lower capability of PCPs (29). This seriously affects the provision of basic health services for the entire population and poses a major challenge for the Chinese government’s development strategy for healthy ageing. This study aimed to understand the overall level of geriatric healthcare service capability of PCPs in China, to provide recommendations for solving the problem, and to promote the goal of healthy ageing.

## 2 Methods

### 2.1 Design

The study was based on a survey conducted in August 2020. It was reviewed and approved by the ethics committee of Nanjing Medical University (NMU-ER (2020) 589). The survey was explained to all participating PCPs as being for research purposes only and as not related to personal evaluation. The questionnaire was anonymous and filled out voluntarily to ensure the objectivity of the self-assessment. Respondents were, though, asked to leave their contact telephone numbers for reviewing the questionnaire.

### 2.2 Sampling and participants

The survey used a combination of multi-stage stratified cluster sampling and typical sampling of PCPs in the eastern, central, and western regions of China. First, two provinces in each of the three regions were selected as sample provinces that were better at the tiered healthcare system construction and displayed certain characteristics relevant to the strategy of improving the capability of primary healthcare services. Second, two prefectural cities in each province, with medium levels of economic development and primary health organization constructions were selected. For each city, one municipal district and one county were selected according to the same criteria. All doctors in all primary health organizations—community health service centers (stations), township health centers, and village clinics—in each sample district/county were included in the survey. Due to restrictions around COVID-19, only one province in the east was investigated, and the sample size doubled.

### 2.3 Data collection

A self-assessment questionnaire of family doctors’ individual capability was compiled according to the Indicator System of Family Doctors’ Health Service Capability developed by the research group. The researchers used the Questionnaire Star platform (the most widely used online questionnaire filling platform in China) to send the questionnaire to PCPs. Survey content included basic information on PCPs (age, education, professional qualifications, and so on) and a self-evaluation of job abilities. The level of job abilities included two options: “possessing” and “not possessing.” The demand for this capability in the workplace included two options: “needing” and “not needing.” The study mainly analyzed the necessary and relevant competencies required by PCPs to carry out geriatric healthcare services, including capability to diagnose and treat common diseases, to manage the health of older adult people, and to manage the health of patients with chronic diseases. A total of 8,469 valid questionnaires were collected from PCPs.

### 2.4 Data analysis

#### 2.4.1 Professional qualifications

Professional qualification categories in the questionnaire were multiple-choice to reduce the potential for errors if PCPs added professional qualifications. Professional qualifications were divided into four categories: licensed (assistant) doctors, GPs, licensed village doctors (LVDs), and others or those without qualifications.

LVD refers to a doctor with a rural doctor practice qualification, usually issued by the health department at or above county level in China. Doctors holding this certificate can legally engage in primary healthcare at village clinics, including disease diagnosis, issuing prescriptions, and treating common diseases.

Some PCPs who hold licensed (assistant) doctor status have also obtained the qualification certificate for GP transfer training, or have added a GP qualification and so can practice as a GP. Therefore, if a PCP chose both “licensed (assistant) doctor” and “GP” or only “GP,” they were classified as “GP.”

#### 2.4.2 Criteria for judging capability

We selected three elements from the Self-Assessment Questionnaire for Family Physicians’ Competencies for analysis: diagnosis and treatment of common diseases, health management of older adult people, and health management of patients with chronic diseases. A judgement was made about whether or not the capabilities were possessed of PCPs, based on the mastery of specific skills for each capability. In addition, the need of PCPs for the capability in the workplace was judged according to their demand for specific skills for each capability.

##### 2.4.2.1 The capability to diagnose and treat common diseases

The capability to diagnose and treat common diseases involves identifying diseases, interpreting common hospital inspection and test reports, and prescribing common medications. PCPs could be judged to basically have skills in these areas if they achieved two out of three items. For example, identifying and diagnosing common diseases mainly focuses on the six most common diseases (respiratory infection, diarrhea, hypertension, diabetes, stroke, and COPD) in primary health services. If the PCP could diagnose and identify more than two out of three (four or more diseases in this case), they were judged to have mastered this skill. And a PCP is considered to have the capability to diagnose and treat common diseases when they have mastered all three skills.

The same two out of three approaches were used for estimating whether the skills were needed in the work. For diagnosing and treating the six diseases mentioned above, PCPs were asked whether this capability was required in daily work. The skill was deemed to be needed if four or more were required (see Table 1).

**Table 1:** Indicator system for evaluating the older adult healthcare service capabilities of PCPs.

Capability	Skill	Skill items
Capability to diagnose and treat common diseases	Identification and diagnosis of common diseases	1. Respiratory infection 2. Diarrhea 3. Hypertension 4. Diabetes 5. Stroke 6. COPD
	Interpretation of common hospital inspection and test reports	1. Three major routines (blood routine, urine routine, stool routine) 2. Ultrasound 3. X-ray examination 4. Biochemical examination report 5. Electrocardiogram examination report
	Prescription of common medications	1. Antibiotics 2. Common cold and flu medicines 3. Chronic disease medicines 4. Emergency medicines
Capability to manage the health of the older adult	1. Physical examination guidance and screening for common diseases of the older adult	
	2. Assessing the health and self-care capability of the older adult	
	3. Identifying of and intervention for low mood or depression	
	4. Prevention of functional deterioration of the older adult	
Capability to manage the health of patients with chronic diseases	1. Mastering the clinical diagnostic criteria for common chronic diseases	
	2. Mastering the major risk factors and intervention methods for common chronic diseases	
	3. Mastering the identification and management of critical conditions in common chronic diseases	
	4. Mastering the treatment of stable chronic diseases and the prevention of complications	

##### 2.4.2.2 The capability to manage the health of the older adult

Older adult health management competencies include four specific skills: physical examination guidance and screening for common diseases, assessment of health and self-care capability, identification of and intervention for low mood or depression, and prevention of functional deterioration. Mastery of three or more of these skills is judged to be a basic mastery of the capability (see Table 1).

##### 2.4.2.3 The capability to manage the health of patients with chronic diseases

Chronic diseases are the most common health problems in the older adult population. Health management of patients with chronic diseases is also one of the most basic service contents of older adult health services. The health management capability of patients with chronic diseases includes mastering the clinical diagnostic basis of common chronic diseases, mastering the main risk factors and intervention methods of common chronic diseases, mastering the identification and treatment methods of common chronic diseases, and mastering the treatment of common chronic diseases during the stabilization period and the prevention of complications in four specific skills. Mastery of three or more skills is judged to be a basic mastery of health management of patients with chronic diseases. See Table 1.

#### 2.4.3 Analysis method

We conducted statistical analysis on the data by using SPSS 28.0, where all count data were expressed as frequency and percentage. Descriptive statistical analysis was used, and the chi-square test was used for inter-group comparison. A two-tailed *α* < 0.05 was considered statistically significant.

## 3 Results

There is an imbalance in the level of economic development among the east, middle, and west of China. It can be observed that the eastern region has the most developed economy, while the western region is relatively backward. Such differences may affect the staffing of PCPs, as medical resources are often correlated with the level of economic development in the region. Therefore, in the comparative analysis, we mainly divided China into three regions: east, central, and west, and focused on the basic characteristics of PCPs and their capability to provide geriatric healthcare services.

### 3.1 Demographic

Of the PCPs who participated in the study, 2,953 (34.9%), 3,200 (37.8%), and 2,316 (27.3%) were from the eastern, central, and western regions, respectively. They were mainly 30 to 44 years old (44.5%) and 4.

Five to 60 years old (42.6%), with 8.8% under 30, and 4.1% over 60. The average age was 43.2. In terms of education, 38.5% of PCPs were below high school/secondary school level, 33.9% had an undergraduate education or above (33.9%), and 27.6% had a junior college education. In terms of professional qualifications, licensed doctors or licensed assistant doctors accounted for 62.4%, followed by LVDs at 26.0, 6.3% had other or no professional qualifications, and 5.3% had GP qualifications.

It is worth noting that, in China, medical educational programs include five- to eight-year post-high school training programs, three-year post-high school programs (which are decreasing), four-year post-middle school programs (which have almost disappeared), and “barefoot” doctors (who tend to be farmers with usually just three to 6 months of basic medical training), who take care of the primary healthcare needs in their communities.

Comparing the regions, we found PCPs in the eastern region mainly had undergraduate education or above, PCPs in the central region mainly had reached the level of technical secondary school/high school or below, and PCPs in the western region mainly had a college degree. The top two types of professional qualification in each region were licensed (assistant) doctor and LVD, and the proportion of GPs was relatively low. The differences in age, education, and professional qualifications of PCPs in different regions were statistically significant (*p* < 0.05) (Table 2).

**Table 2:** Basic demographics of PCPs in the eastern, central, and western regions.

	Eastern	Central	Western	Total
Total number	2,953	3,200	2,316	8,469
Age group (years)**				
<30	294 (10.0)	188 (5.9)	260 (11.2)	742 (8.8)
30-	1,327 (44.9)	1,414 (44.2)	1,029 (44.4)	3,770 (44.5)
45-	1,234 (41.4)	1,438 (44.9)	945 (40.8)	3,607 (42.6)
60-	108 (3.7)	160 (5.0)	82 (3.5)	350 (4.1)
Education level**				
Technical secondary school/ high school or below	869 (29.4)	1,637 (51.2)	756(32.6)	3,262(38.5)
Junior college	633 (21.4)	921 (28.8)	786 (33.9)	2,340 (27.6)
Undergraduate and above	1,451 (49.1)	642 (20.1)	774 (33.4)	2,867 (33.9)
Professional qualification**				
Licensed doctor or licensed assistant doctor	2,209 (74.8)	1,793 (54.3)	1,340 (57.9)	5,288 (62.4)
General practitioner	109 (3.7)	236 (7.4)	101 (4.4)	446 (5.3)
Licensed village doctor	365 (12.4)	1,115 (34.8)	721 (31.1)	2,201 (26.0)
Other or no professional qualifications	270 (9.1)	110 (3.4)	154 (6.6)	534 (6.3)

** *P*<0.01.

### 3.2 Capability possessed for geriatric health services

#### 3.2.1 Capability possessed for diagnosis and treatment of common diseases

The proportion of PCPs in China with the capability to diagnose and treat common diseases was 76.7%, with a difference in the rate of capability in the eastern, central, and western regions (*χ*^2^ = 148.439, *p* < 0.05). The rate was highest in the central region (80.9%), followed by the eastern region (79.3%), and lowest in the western region (67.7%). The capability possessed in urban and rural areas was 76.3 and 76.9%, respectively (*χ*^2^ = 0.451, *p* = 0.502) (Table 3).

**Table 3:** Capability possessed of geriatric healthcare services.

Capability possessed	Region	Urban		Rural		Total	
		Respondents	Possessed	Respondents	Possessed	Respondents	Possessed
Diagnosis and treatment of common diseases	Eastern	1,242	930 (74.9)	1,711	1,412 (82.5)	2,953	2,342 (79.3)
	Central	1,097	880 (80.2)	2,103	1,708 (81.2)	3,200	2,588 (80.9)
	Western	5,24	374 (71.4)	1,792	1,193 (66.6)	2,316	1,567 (67.7)
	Total	2,863	2,184 (76.3)	5,606	4,313 (76.9)	8,469	6,497 (76.7)
Health management	Eastern	1,242	1,099 (88.5)	1,711	1,567 (91.6)	2,953	2,666 (90.3)
	Central	1,097	993 (90.5)	2,103	2,032 (96.6)	3,200	3,025 (94.5)
	Western	524	455 (86.8)	1,792	1,609 (89.8)	2,316	2,064 (89.1)
	Total	2,863	2,547(89.0)	5,606	5,208 (92.9)	8,469	7,755 (91.6)
Patients with chronic diseases	Eastern	1,242	1,102 (88.7)	1,711	1,590 (92.9)	2,953	2,692 (91.2)
	Central	1,097	992 (90.4)	2,103	2,023 (96.2)	3,200	3,015 (94.2)
	Western	524	469 (89.5)	1,792	1,611 (89.9)	2,316	2,080 (89.8)
	Total	2,863	2,563 (89.5)	5,606	5,224 (93.2)	8,469	7,787 (91.9)

#### 3.2.2 Capability possessed for health management

The proportion of PCPs in China with the capability to provide health management services for older adult people was 91.6%, with a difference in the capability of PCPs from eastern, central, and western regions (*χ*^2^ = 60.723, *p* < 0.05). The central region had the highest capability of PCPs, at 94.5%, and the western region had the lowest rate of PCPs, at 89.1%. The capability of PCPs in rural areas was higher than in urban areas (*χ*^2^ = 38.066, *p* < 0.05) (Table 3).

#### 3.2.3 Capability possessed for patients with chronic diseases

The proportion of PCPs in China with the ability to provide health management services for patients with chronic diseases was 91.9%, and there was a difference in the ability rate of PCPs in the eastern, central, and western regions (*χ*^2^ = 39.048, *p* < 0.05). The capability rate of PCPs in the central region was the highest, 94.2%, and the ability rate in the western region was the lowest, 89.8%. The capability of PCPs in rural areas was higher than in urban areas (*χ*^2^ = 34.368, *p* < 0.05) (Table 3).

### 3.3 Capability possessed for older adult healthcare by characteristics of PCPs

The survey showed differences in the capability possessed rates of PCPs with different academic qualifications in terms of diagnosing and treating common diseases, managing the health of the older adult, and managing the health of patients with chronic diseases (*p* < 0.05). In terms of the capability to diagnose and treat common diseases, the capability possessed rate of doctors with undergraduate education or above was higher than that of PCPs with high school education or below (*p* < 0.05); As for health management capability for both the older adult and for patients with chronic diseases, the lower the education level, the higher the rate of capability (*p* < 0.05).

There were differences between PCPs with different professional qualifications in the capability to diagnose and treat common diseases, and for managing the health of the older adult and patients with chronic diseases (*p* < 0.05). The capability rate of licensed (assistant) doctors was higher than that of LVDs (*p* < 0.05) in terms of the capability to diagnose and treat common diseases. The capability rate of GPs and LVDs was higher for managing the health of the older adult and patients with chronic diseases (Table 4).

**Table 4:** Capabilities for geriatric healthcare services by characteristics of PCPs.

	Respondents	Capability to diagnose and treat common diseases	Capability for health management of the older adult	Capability for health management of patients with chronic diseases
Education level**				
Technical secondary school/ high school or below	3,262	2,441 (74.8)	3,116 (95.5)	3,086 (94.6)
Junior college	2,340	1,753 (74.9)	2,129 (91.0)	2,118 (90.5)
Undergraduate and above	2,867	2,303 (80.3)	2,510 (87.5)	2,583 (90.1)
Professional qualification**				
Licensed doctor or licensed assistant doctor	5,288	4,316 (81.6)	4,781 (90.4)	4,873 (92.2)
General practitioner	446	351 (78.7)	433 (97.1)	427 (95.7)
Licensed village doctor	2,201	1,569 (71.3)	2,115 (96.1)	2,084 (94.7)
Other or no professional qualifications	534	261 (48.9)	426 (79.8)	403 (75.5)
Total	8,469	6,497 (76.7)	7,755 (91.6)	7,787 (91.9)

** *P*<0.01.

### 3.4 Skills possessed and needed in the geriatric healthcare service capability of PCPs

The survey showed that for the 11 specific skills of geriatric healthcare services, the possession rate of PCPs had not reached 95%. The top three for the rate of capability were guidance around physical examinations for the older adult and screening for common diseases, diagnosis and identification of common diseases, and clinical diagnosis of chronic diseases. The lowest rate was for the capability to interpreter common hospital inspection and test reports––a possession rate of 81.9%. For the 11 skills, except for the skill of interpreting common hospital inspection and test reports, more than 90% of the PCPs indicated they needed to use the skills in their daily work. For the skill of interpreting common hospital inspection and test reports, although the percentage of doctors who needed this skill in their daily work was 86.5% (slightly lower than the need for the other skills), it was still higher than the rate of PCPs who had actually mastered this skill (Table 5).

**Table 5:** Skills possessed and needed in the geriatric healthcare service capability of PCPs.

Capability	Skill	Skill possessed	Skill needed
Capability in diagnosing and treating common diseases	Identification and diagnosis of common diseases	8,019 (94.7)	7,713 (91.1)
	Interpretation of common hospital inspection and test reports	6,938 (81.9)	7,322 (86.5)
	Prescription of common medications	7,686 (90.8)	7,745 (91.5)
Capability for health management of the older adult	Physical examination guidance and screening for common diseases of the older adult	8,035 (94.9)	7,854 (92.7)
	Assessing the health and self-care capability of the older adult	7,938 (93.7)	7,796 (92.1)
	Identification and intervention of low mood and depression of the older adult	7,715 (91.1)	7,676 (90.6)
	Prevention of functional deterioration of the older adult	7,633 (90.1)	7,636 (90.2)
Capability for health management of patients with chronic diseases	Mastering the clinical diagnostic criteria for common chronic diseases	7,991 (94.4)	7,875 (93.0)
	Mastering the major risk factors and intervention methods for common chronic diseases	7,887 (93.1)	7,844 (92.6)
	Mastering the identification and management of critical conditions in common chronic diseases	7,728 (91.3)	7,770 (91.7)
	Mastering the treatment of stable chronic diseases and the prevention of complications	7,771 (91.8)	7,768 (91.7)

## 4 Discussion

The Chinese government’s key strategy for coping with an ageing society is to establish an ageing service system that focuses on home-based care. This is not only a need based on the emotional well-being of older adult people and their families, but also a development trend in the international community (30). According to the above analysis results, overall, although there are some differences in the quality of PCPs and their capability to provide older adult healthcare services in the eastern, central and western regions of China, but the differences are not as significant as the economic differences in the three regions. It can be seen that the Chinese government has made a lot of efforts to promote the equity in health resource allocation and has achieved certain results.

Our study showed that China’s PCP workforce was generally weak. We found that PCPs have a low level of education, with only 33.9% of them having completed undergraduate education or above. PCPs are also ageing, with 46.7% over 45 years. There is also a lack of GPs––just 5.3% of the PCPs surveyed. There are certain differences in levels of staff in the three regions, with PCPs in the central region having a low level of education, and the proportion of licensed (assistant) doctors in the eastern region being relatively high. In addition, although the economic level of the western region is the lowest among the three regions, the proportion of licensed (assistant) doctors is not the smallest. Mainly because after deepening the reform of the healthcare system, the Chinese government has strengthened its preferential healthcare policies towards the western regions.

To ensure the supply of healthcare services for older adult people, the Chinese government has put forward a series of policies to promote training, better utilization, and offer incentives for PCPs (31). For example, the Chinese government has adopted a policy of free targeted training for students in medical schools, sending the trained students to grassroots practice, especially to rural areas. There are also policies for promotion and career development that aim to help PCPs optimize their career development paths (32–34).

Overall, our study found that the capability of Chinese PCPs to provide geriatric healthcare services needed to be strengthened. The percentage of PCPs with the capability to diagnose and treat common diseases was 76.7%, which is significantly lower than the capability to manage the health of older adult and chronic disease patients. In terms of specific skills, the skill to diagnose and identify common diseases, as well as the skill to apply commonly used drugs, are relatively good. However the percentage of PCPs with the skill of interpreting common hospital inspection and test reports was 81.9%, which was significantly lower than the percentage of PCPs who needed this skill in their daily work (86.5%). A study in India found that training physicians in interpreting chest X-rays significantly improved their capability to interpret chest X-rays. This may provide some reference for improving PCPs’ skills in interpreting common hospital inspection and test reports (35).

The rates of PCPs’ capability to provide health management services for the older adult and for patients with chronic diseases were 91.6 and 91.9%, respectively. With the approach of integrated care for older adult people, they and chronically ill patients usually need more frequent health monitoring, disease screening, and prevention services. With capability in this area at less than 95%, a number of PCPs are unable to provide basic health management services in this regard. However, in reality, this should be their main job responsibility. A survey in Germany analyzed the main reasons that older adult people consulted a GP and found that the top reason was the follow-up treatment of common cardiovascular and endocrine diseases (36). This highlights the importance of health management services for older adult people and for those with chronic conditions. The Chinese government needs to make further efforts to improve the skills of health management-related services to satisfy the increasing demand for healthcare services.

We found differences in the geriatric healthcare service capabilities of PCPs in different regions of China and between urban and rural areas. PCPs in the western region had the lowest rates of all geriatric service competencies, while the central region had the highest rates. In recent years, China has strengthened the primary healthcare workforce in the western region (37), and the proportion of PCPs under 30 in the western region is relatively high and the educational level of PCPs has increased. However, the actual geriatric healthcare capability has not increased accordingly, which may be related to the lack of experience of primary healthcare services in newly graduated PCPs. The proportion of PCPs with undergraduate education or above is the lowest in the central region, and the proportion of those with high school education or below is the highest. However, those with a high school education or below are often more experienced PCPs who have been engaged in grassroots work for a long time. At the same time, older adult healthcare services can be practical, and those with practical experience are more likely to excel in the work. Therefore, the service capability of PCPs in the central region is better than in other regions.

Our survey also showed that, in addition to the diagnosis and treatment of common diseases, PCPs in rural areas had a better capability to manage the health of older adult people and of patients with chronic diseases than those in urban areas. There are more older adult people in rural areas who tend to have a more stable relationship with their PCPs, therefore, PCPs has more opportunities to interact with the older adult in rural areas and gain rich practical experience (38). As a result, PCPs in rural areas show a higher proportion of capability.

Our findings lead us to believe that attention needs to be paid to the development of basic skills in geriatric healthcare services for PCPs. In terms of healthcare management for older adult people and those with chronic conditions, less-educated PCPs have been shown to have higher capabilities across the range Since PCPs mainly focus on managing common health problems and health guidance in geriatric healthcare services (38), and less-educated PCPs are usually LVDs who have more practical opportunities and experience, so their mastery of frequently used knowledge and skills tends to be better (39). The results also show that there is a high need for PCPs to have the full range of geriatric healthcare skills––the need rate for 10 of the 11 skills exceeded 90%.

The structure of the PCP workforce in China is unlikely to change in the near future, so training in geriatric healthcare-related skills and in improving the mastery of commonly used knowledge and skills should be a key strategy for the development of PCPs and the integrated healthcare service for older adult people.

## 5 Limitations

Our questionnaire was based on the PCPs’ self-judgment of PCPs’ individual capabilities rather than objective measurements, so the results may not always match the actual situation. And currently, the self-judgment of the older adult’s health service capabilities and needs has only been conducted from the perspective of PCPs. The next step is to further evaluate the level and needs of PCPs’ older adult health service capabilities from the demand side, and provide a multi-dimensional basis for improving the older adult health service capabilities.

## 6 Conclusion

The policy of integrating eldercare services with medical care aims to meet the needs of older adult people for both health and ageing. PCPs are the main providers of healthcare services for older adult people. Our study found that China’s PCP workforce is generally weak and there are deficiencies in all aspects of geriatric healthcare services. The capability of PCPs varies across regions and between urban and rural areas. China’s PCP workforce should be strengthened with targeted training and the development of basic skills for PCPs in geriatric healthcare services. Firstly, it is imperative to establish and refine a comprehensive training mechanism, providing appropriate training content and formulating supplementary policies to support PCPs’ training. Targeted training programs should be implemented, focusing on various competencies in older adult healthcare service projects, particularly addressing existing skill gaps. Secondly, given the strong practical nature of various older adult healthcare service projects, practical skills can be further enhanced through organizing older adult healthcare service skills competitions and conducting practical drills. Thirdly, all competencies involved in older adult healthcare services should be incorporated into the PCP assessment system, with regular evaluations and assessments conducted. Simultaneously, corresponding incentive mechanisms should be established to complement these efforts. Additionally, the Chinese government has adopted a policy of free targeted training of students in medical schools, sending the trained students to grassroots practice, especially in rural areas. There are also policies for promotion and career development that aim to help PCPs optimize their career development paths (31–33).

## Data Availability

The original contributions presented in the study are included in the article/supplementary material, further inquiries can be directed to the corresponding authors.
